# Study on the Grooved Morphology of CMC-SiC_f_/SiC by Dual-Beam Coupling Nanosecond Laser

**DOI:** 10.3390/ma15196630

**Published:** 2022-09-24

**Authors:** Tao Chen, Xiaoxiao Chen, Xuanhua Zhang, Huihui Zhang, Wenwu Zhang, Ganhua Liu

**Affiliations:** 1 School of Mechanical and Electrical Engineering, Jiangxi University of Science and Technology, Ganzhou 341000, China; 2 Ningbo Institute of Materials Technology and Engineering, Chinese Academy of Sciences, Zhejiang Provincial Key Laboratory of Aero Engine Extreme Manufacturing Technology, Ningbo 315201, China; 3 University of Chinese Academy of Sciences, Beijing 100049, China; 4 School of Mechanical Engineering and Mechanics, Ningbo University, Ningbo 315211, China

**Keywords:** ceramic matrix composite, dual-beam coupling, nanosecond laser, grooved morphology, high-temperature oxidation

## Abstract

Due to the excellent properties of high hardness, oxidation resistance, and high-temperature resistance, silicon carbide fiber reinforced silicon carbide ceramic matrix composite (CMC-SiC_f_/SiC) is a typical difficult-to-process material. In this paper, according to the relationship between the spatial posture of dual beams and the direction of the machining path, two kinds of scanning methods were set up. The CMC-SiC_f_/SiC grooving experiments were carried out along different feeding directions (transverse scanning and longitudinal scanning) by using a novel dual-beam coupling nanosecond laser, and the characteristics of grooving morphology were observed by Laser Confocal Microscope, Scanning Electron Microscopy (SEM), and Energy Dispersive Spectrometer (EDS). The results show that the transverse scanning grooving section morphology is V shape, and the longitudinal scanning groove section morphology is W shape. The grooving surface depth and width of transverse scanning are larger and smaller than that of longitudinal scanning when the laser parameters are the same. The depth of the transverse grooving is greater than that of the longitudinal grooving when the laser beam is transverse and longitudinal scanning, the maximum grooving depth is approximately 145.39 μm when the laser energy density is 76.73 J/cm^2^, and the minimum grooving depth is approximately 83.76 μm when the laser energy density is about 29.59 J/cm^2^. The thermal conductivity of fiber has a significant effect on the local characteristics of the grooved morphology when using a medium energy density grooving. The obvious recasting layer is produced after the laser is applied to CMC-SiC_f_/SiC when using a high energy density laser grooving, which directly affects the grooved morphology.

## 1. Introduction

Silicon carbide fiber-reinforced silicon carbide ceramic matrix composite (CMC-SiC_f_/SiC) has excellent mechanical properties and stable chemical properties [1]. It has a wide application prospect in the fields of hypersonic aircraft [2], the nuclear energy industry [3], and the national defense industry [4].

Because CMC-SiC_f_/SiC is a difficult-to-process material [5] and laser processing has the advantages of non-contact, greening, and high precision, domestic and foreign scholars have gradually paid attention to laser processing CMC-SiC_f_/SiC. Zhai et al. [6] used a femtosecond laser to etch carbon fiber reinforced silicon carbide ceramic matrix composites (CMC-C_f_/SiC) and found that the laser removal mechanism could be changed from photothermal effect to photochemical effect by changing the laser parameters. Moreover, Zhai et al. [7,8] found through further research that the femtosecond laser refracts and scatters on the surface of the material when the surface of the material is uneven, resulting in the narrowing or bending of the microgrooves. Nasiri et al. [9] think that the oxidation process of CMC-SiC_f_/SiC was related to temperature and time. The differences in the structure of fiber composites and the properties of each component affect the laser removal mechanism of fiber composites and the surface morphology [10]. Zhang et al. [11] used a millisecond laser to ablate CMC-SiC_f_/SiC and found that the ablation area has problems such as fiber fracture, interface–matrix debonding, interface–fiber debonding, and fiber hollowing. The evolution mechanism is closely related to the internal stress caused by the temperature gradient in the ablation region and the melting temperature difference of the material components. Wei et al. [12] studied the removal mechanism of CMC-SiC_f_/SiC by underwater femtosecond laser ablation and found that the removal mechanism of CMC-SiC_f_/SiC was the decomposition of SiC; they believe that the SiC matrix was first removed, and the interface layer was then removed, and the SiC fibers were finally removed. Du et al. [13] ablated CMC-SiC_f_/SiC with oxygen-acetylene flame and found that the material removal mechanism is dominated by the sublimation of the matrix and fibers, and the ablation process is accompanied by mechanical spalling and material oxidation. Liu et al. [14] used millisecond laser processing of CMC-SiC_f_/SiC and found that three kinds of tiny particles with different diameters were attached to the surface of the recasting layer, spherical micro protrusion (20–48 μm), bubble particle (5–15 μm), and submicron particles (<1 μm), respectively. Jiao et al. [15] used a nanosecond laser to remove CMC-C_f_/SiC and found that heat accumulation was the main factor in removing CMC-C_f_/SiC; in addition, the heat was mainly transferred along the fiber arrangement direction.

In addition to studying the mechanism of laser removal of ceramic matrix composite (CMC), scholars also analyzed the characteristics of different types of laser-processed fiber composite materials. Gavalda Diaz et al. [16] suggested that various types of lasers to remove CMC would generate heat affected zones (HAZ), but the width of the HAZ could be effectively reduced by using a laser with shorter pulse width. Takahashi et al. [17] used Infrared (IR) and Ultraviolet (UV) lasers to groove carbon fiber reinforced plastic (CFRP) and found that UV laser irradiation produced a narrower HAZ with better processing quality compared to IR lasers. Therefore, the width of the HAZ can be reduced, and higher processing quality can be obtained by selecting the laser parameters [18].

Domestic and foreign scholars have reported on beam-coupling laser removal materials. Chen et al. [19] proposed a new method for machining stainless steel using using the dual-beam coupling nanosecond laser. Zhou et al. [20] coupled continuous laser and pulsed laser at one point to polish S136H die steel with a free-form surface and obtained lower surface roughness. Jiang et al. [21] used a three-beam coupling laser for cladding experiments and achieved better results. Liu et al. [22] established a mathematical model of the relationship between the three-beam coupling laser cladding process parameters and the surface morphology of the cladding layer and obtained a cladding layer with better surface morphology. Therefore, coupling laser processing has unique advantages.

In this paper, CMC-SiC_f_/SiC grooves were carried out along different feed directions by using a new dual-beam coupling nanosecond laser in an air environment. The mechanism of CMC-SiC_f_/SiC dual-beam coupling nanosecond laser grooving was analyzed based on the study of the change in the morphology characteristics of laser grooving. This work expanded the process types of CMC-SiC_f_/SiC laser processing, which can provide technical support for high-performance applications of CMC.

## 2. Materials and Methods

**Figure 1:**
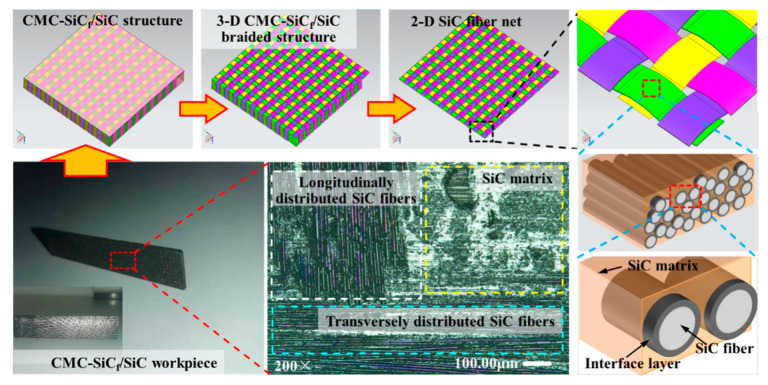
The structure and the surface morphology of CMC-SiC_f_/SiC.

The CMC-SiC_f_/SiC workpiece (100 mm × 20 mm × 2 mm) was used for the dual-beam coupling nanosecond laser grooving experiments. The CMC-SiC_f_/SiC workpiece is composed of SiC fiber, SiC matrix, and interface layer; SiC fiber is arranged in vertical and horizontal weaving manner to form a fiber preform, the interface layer is wrapped on the surface of the SiC fiber, and the CMC-SiC_f_/SiC workpiece is prepared by chemical vapor infiltration (CVI) process, as shown in Figure 1. The diameter of the SiC fiber is about 12–13 μm, and the thickness of the interface layer is approximately 0.4 μm. The preparation process of CMC-SiC_f_/SiC mainly includes the CVI process, precursor impregnation and pyrolysis (PIP) process, and melt infiltration (MI) process. The advantage of the CVI process is that there is basically no damage to the fibers, while the disadvantage is that the prepared composites have high porosity.

**Figure 2:**
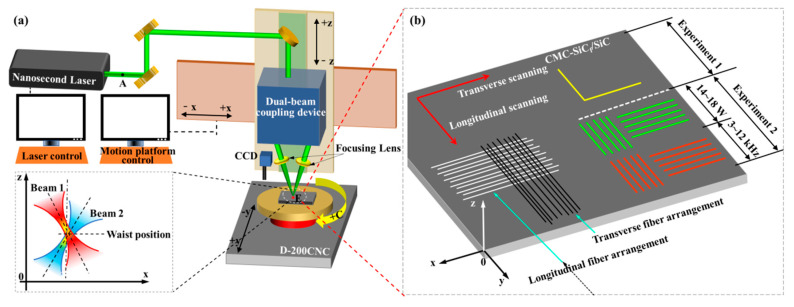
Grooving experimental platform and grooving experimental parameters. (**a**) Laser processing platform; (**b**) grooving experimental parameters.

The schematic diagram of CMC-SiC_f_/SiC is processed by a nanosecond laser, as shown in Figure 2. Processing equipment mainly includes a four-axis motion platform (Model: D-200CNC, China), nanosecond laser, optical platform, optical path system components, control system, etc., as shown in Figure 2a. The pulse width (*τ*) is 78 ns, the wavelength (*l*) is 532 nm, and the beam quality (*M*^2^) less than or equal to 1.5. *M*^2^; is used to describe the quality of the laser beam. The ideal beam quality *M*^2^; is 1, it can be greater than or equal to 1 in practice, and the beam quality of a laser source becomes worse as *M*^2^ increases.

**Figure 3:**
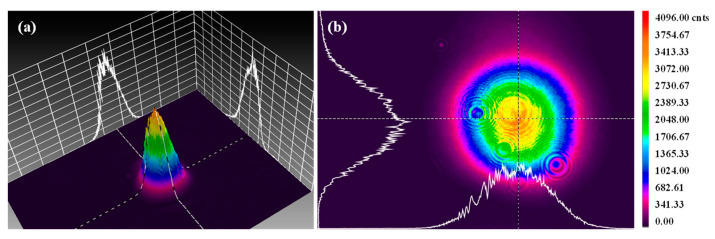
Energy density distribution in the spot of the experimental laser source. (**a**) Three-dimensional energy density distribution; (**b**) two-dimensional energy density distribution.

The spot quality analyzer was placed at point “A” to analyze the beam quality of the laser source, and the laser beam energy density distribution satisfied the Gaussian distribution, as shown in Figure 3.

The single beam was first split into two laser beams, and finally, the two beams were coupled at point “E” by adjusting the angle of the two laser beams. The angles of the two laser beams of the dual-beam coupling nanosecond laser in the y-0-z plane were 1.09 degrees and 1.33 degrees, respectively, and the angle difference between the two beams was 0.24 degrees. The angles in the x-0-z plane are 3.58 degrees and 9.97 degrees, respectively, and the sum of the angles between the two laser beams is 13.55 degrees. Because the focal lengths of the two optical focusing lenses used in the experiment are equal, the two laser beams have the same focal position; that is, the two beams have the same spot size in the beam waist plane of the single beam. However, since the incidence angles of the beams in the x-0-z plane are not equal, the coupled poses of the two laser beams in the x-0-z plane are shown in Figure 2. When the laser pulse energy emitted by the laser source is constant, the pulse energy of the two laser beams is not equal, and the pulse energy of the right laser beam is smaller than the left one.

Fiber composites have high thermal anisotropy in the direction parallel to and perpendicular to the fiber [23]. In the plane, the heat transfer is obvious along the direction parallel to the fiber arrangement, and in space, the heat can be transmitted in multiple directions, including the direction perpendicular to the fiber arrangement [24]. While the substrate absorbs heat, it also transfers heat to the interface layer, which in turn transfers heat to the fiber. In addition to the heat absorbed by the fiber itself from the laser, the fiber also conducts heat from the interface layer. Due to the thermal conductivity of fibers, heat is more easily conducted along the direction of fiber distribution [17].

In order to distinguish the intensity of laser energy density during the double-beam coupling nanosecond laser grooving, the grooving energy region is divided into low energy density grooving, medium energy density grooving, and high energy density grooving. In a certain range of laser energy density, when the grooving track is discontinuous, it is called low energy density grooving, and this range can be called the low energy density region. When the grooving track is continuous, but the recasting layer cannot completely cover the grooving surface, it is called medium energy density grooving, and this interval can be called medium energy density region. When the grooving track is continuous, and the recasting layer completely covers the grooving surface, it is called high energy density grooving, and this interval can be called high energy density region.

Two groups of grooving experiments were carried out in two different regions of the surface of the CMC-SiC_f_/SiC sample: experiments 1 and 2, as shown in Figure 2b. The laser power was measured using the laser power meter at point “E” in Figure 2a. In order to explore the influence of fiber thermal conductivity on grooved morphology, grooving experiment 1 was carried out, as shown in Table 1. In order to explore the influence of different scanning directions of dual-beam coupling nanosecond laser on grooved morphology, experiment 2 was carried out on the basis of grooving experiment 1, as shown in Table 2. In experiment 2, the purpose of changing the laser energy density was achieved by changing the laser power when the laser repetition frequency was 10 kHz, and the laser repetition frequency was changed to achieve the purpose of changing the laser energy density when the laser power was 14 W. The feed speeding (*v*) was 200 mm/min, and the defocus amount was 0 mm in experiments 1 and 2.

The laser energy density is expressed as [25,26]:

(1) $$\varphi =\frac{P}{\pi f\omega_{0}^{2}}$$

where *P* is the average laser power (W), *f* is the laser repetition frequency (Hz), and $\omega_{0}$ is the equivalent waist radius of 44 μm for a dual-beam coupling laser. According to Equation (1), the laser energy densities corresponding to different laser repetition frequencies and laser powers can be calculated as shown in Table 3 and Table 4, respectively.

After the grooving experiments of the two groups, the specimens were immersed in a solution of 99.7% ethanol for 10 min for ultrasonic cleaning, and then the grooved morphology was observed by Laser Confocal Microscope (Model: KEYENCE VK-X200K, Japan), Scanning Electron Microscopy (SEM, Model: QUANTA FEG 250, FEI, Hillsboro, OR, USA 250), and Energy Dispersive Spectrometer (EDS, Model: FEI, Hillsboro, OR, USA).

## 3. Results and Discussions

### 3.1. Analysis of Grooved Topographies of Medium Energy Density Grooving

**Figure 4:**
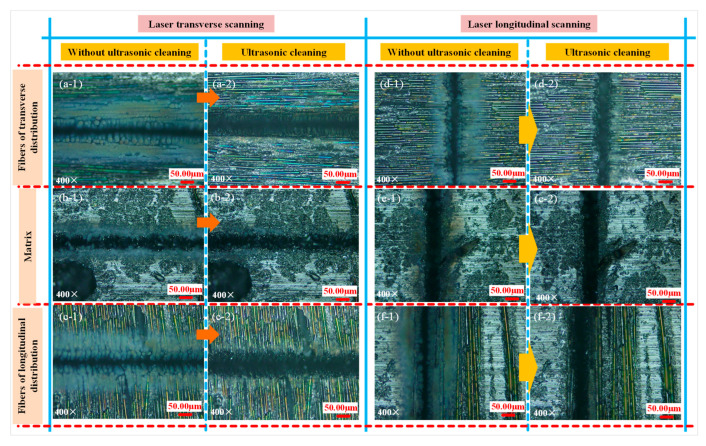
The grooved morphologies of different regions in experiment 1 (**a-1**–**f-2**).

In order to analyze the physical and chemical changes in the surface grooving regions, the grooved morphologies of experiment 1 were observed under laser confocal microscopy, as shown in Figure 4. Under the parameters of experiment 1 (*φ* = 8.22 J/cm^2^), grooves in different scanning directions are continuous. When the laser energy density is 8.22 J/cm^2^, CMC-SiC_f_/SiC can be effectively removed, but the recast layer cannot completely cover the groove surface, which is the grooves with medium energy density. When the nanosecond laser acts on the surface of the material, the high temperature triggers a series of chemical reactions, such as the formation of oxides, and finally, leads to the removal of CMC-SiCf/SiC under the influence of various physical and chemical factors. In the process of nanosecond laser grooving, there is an oxidation phenomenon, and the white solid particles produced are SiO_2_ [27].

**Figure 5:**
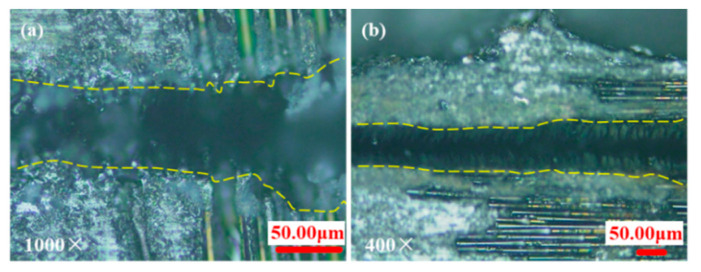
The morphologies of transverse scanning grooves in different regions. (**a**) Regions of matrix and longitudinal fiber arrangement; (**b**) regions of matrix and transverse fiber arrangement.

It was found that the orientation of the fibers could affect the grooved morphology under confocal microscopy, as shown in Figure 5, as it correlates with the thermal conduction velocity of the fibers being higher than that of the matrix and a large difference in the removal temperature of each component of the composite [12,13]. After the light beam is irradiated to the fiber, the fiber can conduct heat along the fiber arrangement direction so that the groove surface morphology of the fiber arrangement area is different from that of the matrix area. Therefore, the orientation and thermal conductivity of the fibers must be considered when processing CMC-SiC_f_/SiC by nanosecond laser at medium energy density.

### 3.2. Analysis of Three-Dimensional Feature of High Energy Density Grooving

#### 3.2.1. Three-Dimensional Feature of Grooves at Different Laser Energy Densities

**Figure 6:**
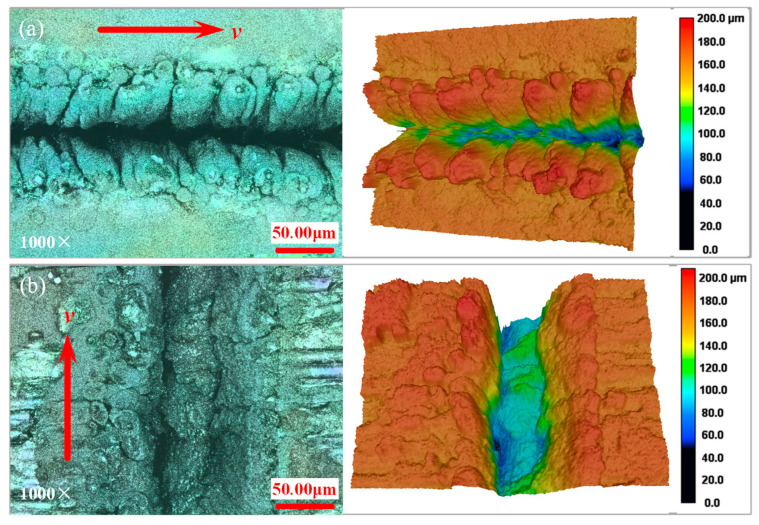
The grooved morphologies in different scanning directions under laser power of 14 W and repetition frequency of 12 kHz. (**a**) Transverse scanning region; (**b**) longitudinal scanning region.

The laser energy density can be increased by increasing laser power and decreasing repetition frequency. It was found that when laser power is 14 W and the repetition frequency is 12 kHz, the energy density *φ* is 19.18 J/cm^2^, and a large number of recasting layers are accumulated on both sides of the grooving surface. CMC-SiC_f_/SiC removed by fiber heat conduction is completely covered by the recasting layer (See Figure 6), which is the high energy density grooving. The depth of the groove bottom on the left is significantly larger than that on the right when the laser is longitudinal scanning, which is caused by the unequal pulse energy of the two laser beams.

**Figure 7:**
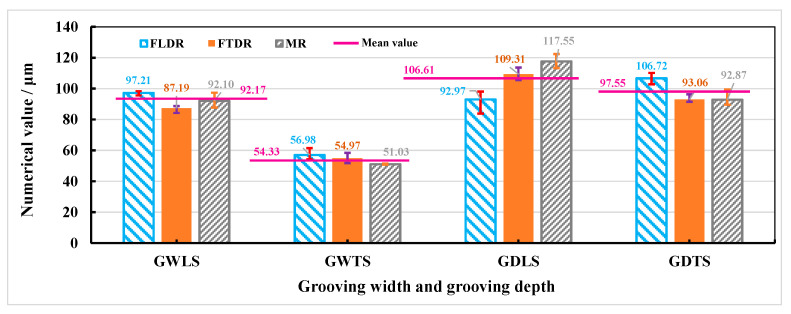
Grooving depth and width in different regions under laser power of 14 W and repetition frequency of 12 kHz.

The values of grooving width of longitudinal scanning (GWLS), grooving width of transverse scanning (GWTS), grooving depth of longitudinal scanning (GDLS), and grooving depth of transverse scanning (GDTS) in the fiber longitudinal distribution region (FLDR), fiber transverse distribution region (FTDR), and matrix region (MR) are shown in Figure 7. It was found that the trough depth and width in each region are close to the overall value, and the error of each set of parameters is small. This is because the recast layer covers the grooved surface, and the width and depth of the groove are mainly affected by the morphology of the recasting layer, while the second factor is the thermal conductivity of the fiber. That is, when the energy density *φ* is greater than or equal to 19.18 J/cm^2^, the morphology of CMC-SiC_f_/SiC dual-beam coupling nanosecond laser etching is less affected by the thermal conductivity of fibers. The recasting layer produced by laser on CMC-SiC_f_/SiC is obvious, which directly affects the grooved morphologies.

**Figure 8:**
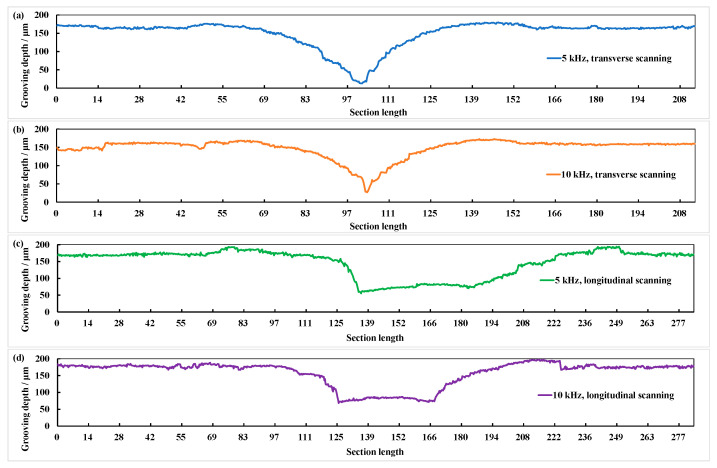
The section morphologies of grooves in different scanning directions. (**a**) Transverse scanning and repetition frequency of 5 kHz; (**b**) transverse scanning and repetition frequency of 10 kHz; (**c**) longitudinal scanning and repetition frequency of 5 kHz; (**d**) longitudinal scanning and repetition frequency of 10 kHz.

Figure 8 shows the grooved section morphologies under different frequencies and scanning directions when laser power is 14 W and defocus amount is 0 mm. It can be seen that the groove shape in transverse scanning is a V shape and that in longitudinal scanning is a W shape. Compared with longitudinal scan grooving, the depth (*h*) and width (*s*) of transverse scan grooving are larger and smaller, respectively. This is because the trajectory of the two laser beams incident from the cross-section of the grooving and acting on the bottom of the grooving almost coincide when the laser beam is transverse scanning. Therefore, a secondary grooving effect in space exists. Two laser beams incident from two directions of the cross-section of the groove and act on both sides of the groove simultaneously when the laser beam is longitudinal scanning. Therefore, there is no secondary grooving effect in space.

**Figure 9:**
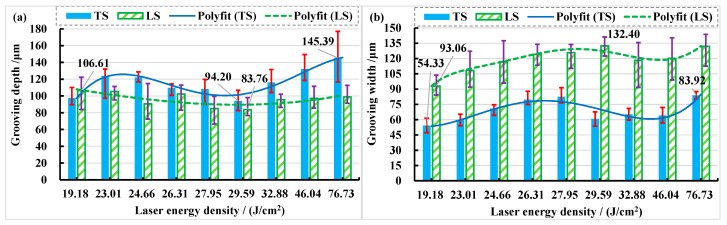
The changes in grooving depth and width in different laser energy densities. (**a**) Grooving depth; (**b**) grooving width.

When the laser beam is transverse scanning, the grooving depth and grooving width show a trend of first increasing, then decreasing, and finally increasing with the increase in laser energy density. When the beam is longitudinal scanning, the grooving depth first decreases and then increases with the increase in laser energy density, while the grooving width first increases, then decreases, and finally increases with the increase in laser energy density, as shown in Figure 9. The depth of the transverse grooving is greater than that of the longitudinal grooving when the laser beam is transverse and longitudinal scanning, the maximum grooving depth is approximately 145.39 μm when the laser energy density is 76.73 J/cm^2^, and the minimum grooving depth is approximately 83.76 μm when the laser energy density is about 29.59 J/cm^2^ (See Figure 9a). The width of the transverse grooving is smaller than that of the longitudinal grooving when the laser beam is transverse and longitudinal scanning; the maximum grooving width is approximately 132.40 μm when the laser energy density is about 29.59 J/cm^2^, the minimum grooving width is approximately 54.33 μm and when the laser energy density is about 19.18 J/cm^2^ (See Figure 9b).

#### 3.2.2. Influence of Different Laser Powers on the Grooved Morphologies

**Figure 10:**
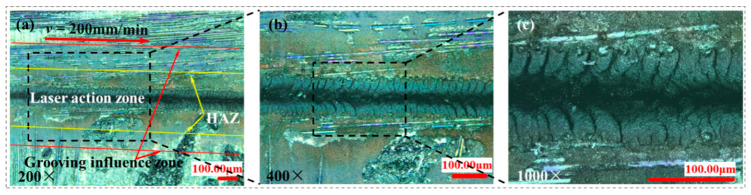
The grooved morphologies with repetition frequency of 10 kHz and laser power of 14 W: (**a**) 200×magnifications; (**b**) 400× magnifications; (**c**) 1000× magnifications.

The grooved morphology is shown in Figure 10 when the repetition frequency is 10 kHz, the laser power is 14 W, the feed speed is 200 mm/min, and transverse scanning. Recasting layers with irregular morphology are generated on both sides of the groove when the energy density is high; this is 23.01 J/cm^2^. This is because when the laser energy density is high enough, CMC-SiCf/SiC melts and is removed. As CMC-SiCf/SiC cools and solidifies in a very short time after the light beam leaves the material irradiation region, it rapidly shrinks irregularly from a molten state to a solid state, forming a recast layer with irregular morphology. Compared with the middle energy density grooves in Figure 5, the recasting layer of high energy density grooves not only covers the grooves completely but also covers some fibers on the material surface. The morphology changes caused by the thermal conductivity of fibers cannot be observed under the confocal microscope at 200×, 400×, and 1000× magnifications. Therefore, it is further indicated that when the laser energy density is high, the obvious recasting layer is produced after the laser is applied to CMC-SiCf/SiC, which directly affects the grooved morphology.

**Figure 11:**
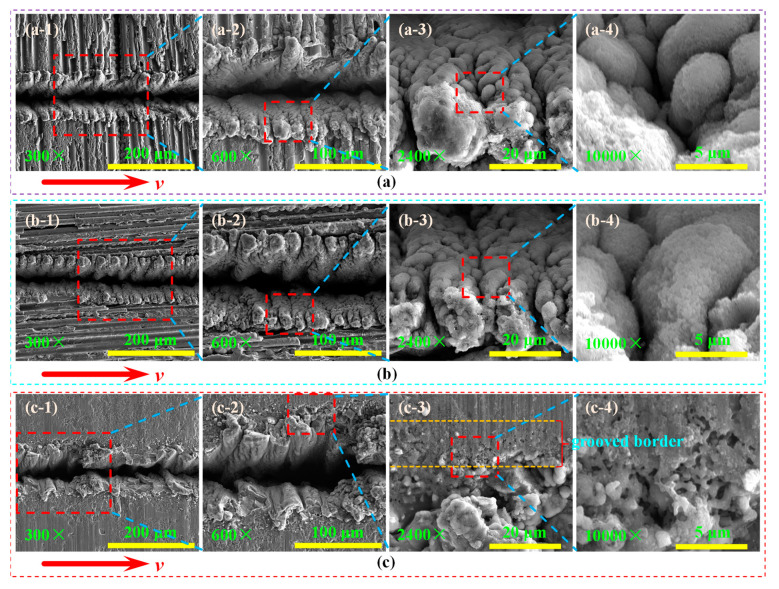
The grooved morphologies in different regions during transverse grooving. (**a-1**–**a-4**) Fiber longitudinal arrangement; (**b-1**–**b-4**) fiber transverse arrangement; (**c-1**–**c-4**) matrix distribution.

When the laser power is 14 W and the repetition frequency is 10 kHz, that is, the energy density in the spot is 23.01 J/cm^2^, the grooved morphologies of laser transverse scanning processing in different regions are shown in Figure 11. The recasting layer completely covers both sides of the grooved surface and presents a “zig-zag” structure along the laser scanning direction, and there are “recessed ravines” on the surface of the recasting layer. The material is in a molten state and is sprayed on both sides of the grooved surface under the condition of high temperature and high pressure. As the temperature and pressure decrease in the laser grooved region, the molten material cools rapidly to form a recasting layer. Under the action of high pressure, the molten material near the laser irradiation sputters around the laser irradiation point and forms a “gully” after cooling. The cooled products are stacked one after another to form a recasting layer. The recasting layer of the grooved surface in the fiber transverse arrangement region and the fiber longitudinal arrangement region presents a flocculent structure (See Figure 11(a-2,b-2)) and is in a fluffy state (See Figure 11(a-4,b-4)). The recasting layer in the matrix distribution region presents a cohesive structure (See Figure 11(c-2)), which is relatively flat compared to the recasting layer in the fiber arrangement region. A small amount of material is removed from the grooved border of the matrix distribution area.

**Figure 12:**
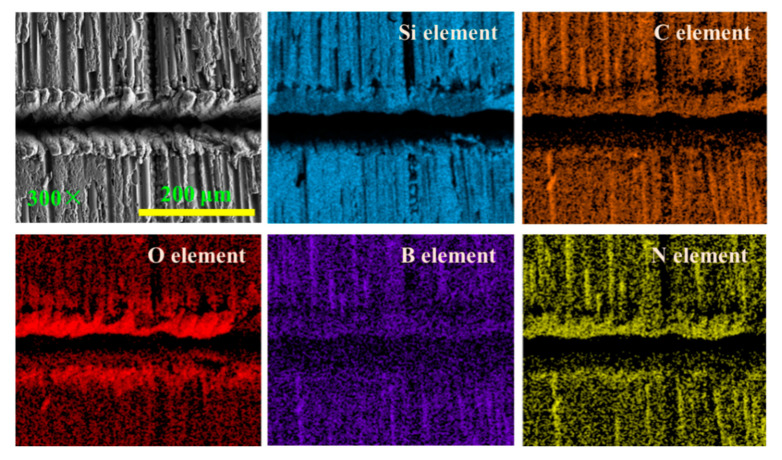
Distribution of different elements of fiber longitudinal arrangement and laser transverse scanning region.

The incidence angle of the two laser beams [28] and the angle between the coupling of the two laser beams can affect the element distribution of the recasting layer. Figure 12 shows the grooved morphology when the longitudinal fiber arrangement and laser transverse scanning region. The distribution of Si, O, N, C, and B element on the surface of the recasting layer on the upper side of the grooved surface is denser than that on the lower side. This is because the laser pulse energy irradiated on the material surface by the laser beam with an oblique angle can be divided into three regions during grooving [25]. The laser energy density on the side with the smaller angle between the laser beam and the material surface is higher, resulting in more oxide distribution of the recasting layer on the upper side of the grooved surface.

**Figure 13:**
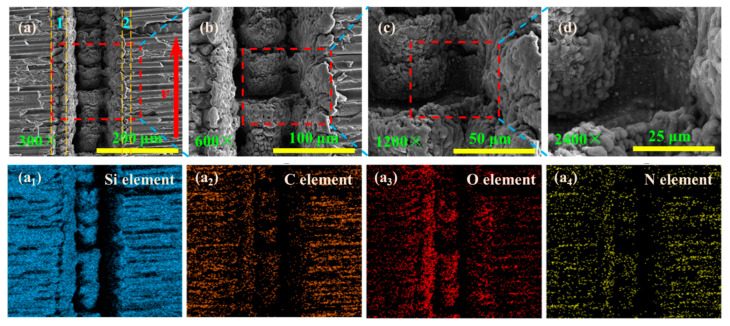
The grooved morphologies corresponding to different magnifications and distribution of different elements of fiber transverse arrangement (**a**_1_–**a**_4_) and laser longitudinal scanning region: (**a**) 300× magnifications; (**b**) 600× magnifications; (**c**) 1200× magnifications; (**d**) 2400× magnifications.

Figure 13 shows the grooved morphology when the fibers are transverse arrangement, and the laser beam is longitudinal scanning. There is a bulge in the center of the grooving, and the recasting layer in the depression in the center of the grooving has cracks under the action of surface tension and residual stress. Because the pulse energy of the two laser beams and the incidence angle in the x-0-z plane are not equal, the distribution of Si, C, O and N elements in the grooved region 1 is denser than that of the grooved region 2. Therefore, the grooved morphology can be changed by changing the pulse energy of the two laser beams and the incidence angle of the laser beams.

**Figure 14:**
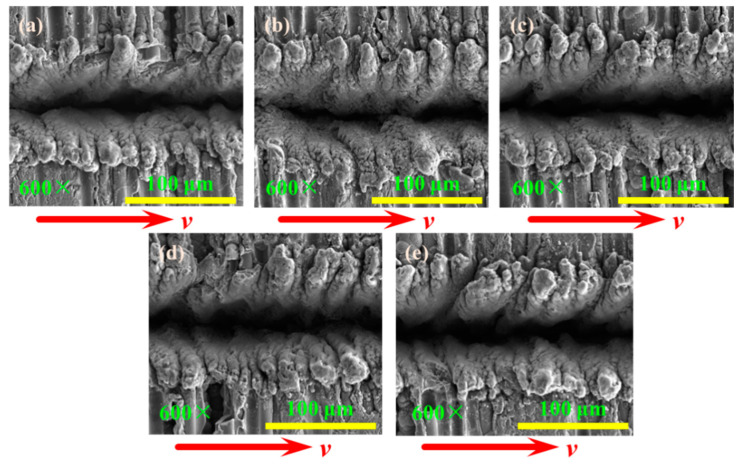
The grooved morphologies corresponding to different laser powers: (**a**) 14 W; (**b**) 15 W; (**c**) 16 W; (**d**) 17 W; (**e**) 18 W.

The grooved morphologies corresponding to different laser powers are shown in Figure 14 when the longitudinal fiber arrangement and laser transverse scanning. The “serrated” tooth profile of the recasting layer becomes more pronounced as the laser power increases. The two laser beams of the dual-beam coupling nanosecond laser are irradiated obliquely on the material surface in the y-0-z plane, and the recasting layer on the upper side of the grooved surface has a more obvious tooth shape.

**Figure 15:**
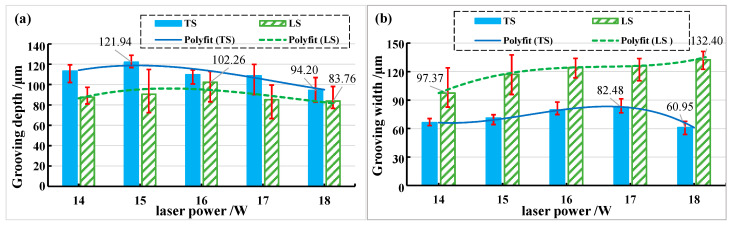
The change in grooving depth and width with increasing laser power in different feeding directions. (**a**) Grooving depth; (**b**) grooving width.

The relationship between grooving depth and grooving width under different laser powers is shown in Figure 15 when the repetition frequency is 10 kHz. It was found that compared with longitudinal scanning (LS), transverse scanning (TS) has a larger depth and smaller width. The grooving depths of the transverse and longitudinal scanning of the laser beam first increased and then decreased with the increase in laser power. This is because the plasma gradually increases with increasing laser power, resulting in a plasma shielding effect [29]. The plasma shielding effect hinders the further action of the laser and CMC-SiC_f_/SiC. However, the amount of recasting layer and oxide generated increases with laser power, and these formations adhere to the grooved surface, ultimately leading to a decrease in groove depth. The grooving width of the transverse and longitudinal scanning of the laser beam first increased and then decreased with the increase in laser power, while the groove width of the longitudinal scanning of the laser beam increased with the increase in the laser power. It can be seen that when the laser beam is transverse scanning, the thickness of the recasting layer covering the grooved surface increases with the increase in laser power, and the thickness of the recasting layer is the largest when the laser power is 18 W, resulting in the smallest grooving width.

#### 3.2.3. Influence of Different Laser Frequencies on the Grooved Morphologies

**Figure 16:**
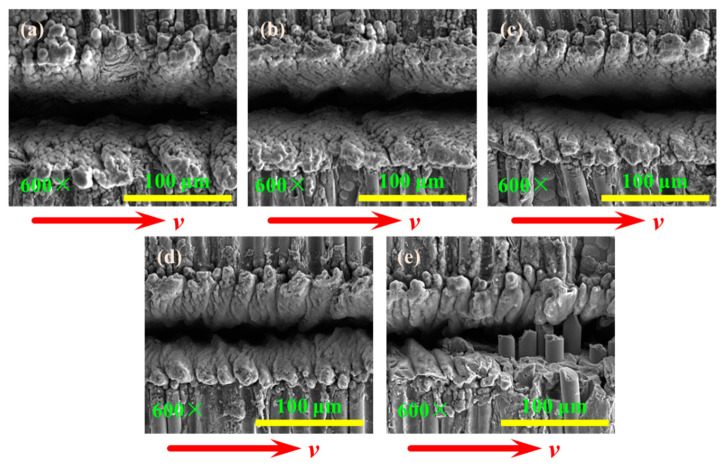
The grooved morphologies corresponding to different repetition frequencies: (**a**) 3 kHz; (**b**) 5 kHz; (**c**) 7 kHz; (**d**) 10 kHz; (**e**) 12 kHz.

The grooved morphologies corresponding to different laser repetition frequencies are shown in Figure 16 when the fiber longitudinal arrangement and laser transverse scanning. The recasting layer of the grooved surface is relatively flat when the repetition frequency is less than 10 kHz. The recasting layer of the grooved surface is more uneven, and the tooth shape is obvious when the repetition frequency is greater than or equal to 10 kHz. The recasting layer of the grooved surface is irregular, and a part of the fibers is not removed when the repetition frequency is 12 kHz.

**Figure 17:**
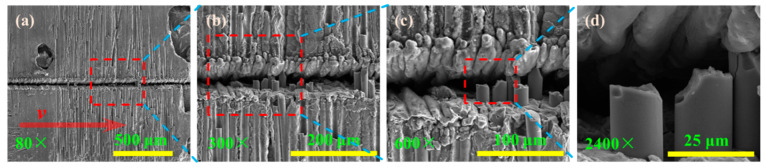
The grooved morphologies corresponding to different magnifications: (**a**) 80× magnifications; (**b**) 300× magnifications; (**c**) 600× magnifications; (**d**) 200× magnifications.

The fibers are cut by the laser beam from the side of the grooved surface, as shown in Figure 17, when the repetition frequency is 12 kHz, laser power is 10 W, fibers are longitudinal arrangement, and laser beam is transverse scanning (*φ* = 19.18 J/cm^2^). This is because the fiber can be removed when the energy density in the laser spot is small, and the laser beam is directly irradiated on fiber, but the reflected laser cannot remove the fiber. Compared to SiC matrix, SiC fibers are more difficult to be removed by laser.

**Figure 18:**
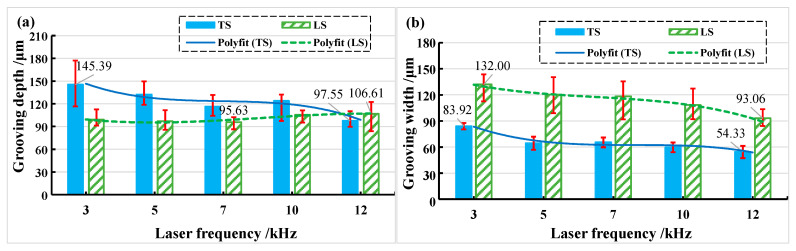
The change in grooving depth and width with increasing frequency in different feeding directions. (**a**) Grooving depth; (**b**) grooving width.

The relationship between grooving depth and grooving width at different repetition frequencies is shown in Figure 18 when the laser power is 14 W. It was found that transverse scanning (TS) has greater depth and narrower width than longitudinal scanning (LS). When the frequency is 3 kHz, the measurement deviation of transverse scanning depth is large. This is because the repetition frequency is smaller so that the energy density in the unit area is more concentrated, the number of pulses at the same time is smaller, and the spacing and depth of pulse points at the bottom of the tank are larger. The fluctuation range of measured values increases as the degree of unevenness increases (See Figure 5a). Longitudinal scanning of the grooved morphology shows that the depth on both sides of the bottom of the grooving is not equal (See Figure 5b). This is because when the beam irradiates to the bottom of the grooving, it is already in a positive defocusing state, and the energy density of the two laser beams from the beam splitter is not equal.

## 4. Grooving Mechanism

The above analysis can provide theoretical support for the study of the CMC-SiC_f_/SiC dual-beam coupling nanosecond laser grooving process. In the process of nanosecond laser grooving, the thermal effect plays a major role and forms the laser action region. Heat diffuses from the center of the laser action area to the periphery to form the HAZ [18].

**Figure 19:**
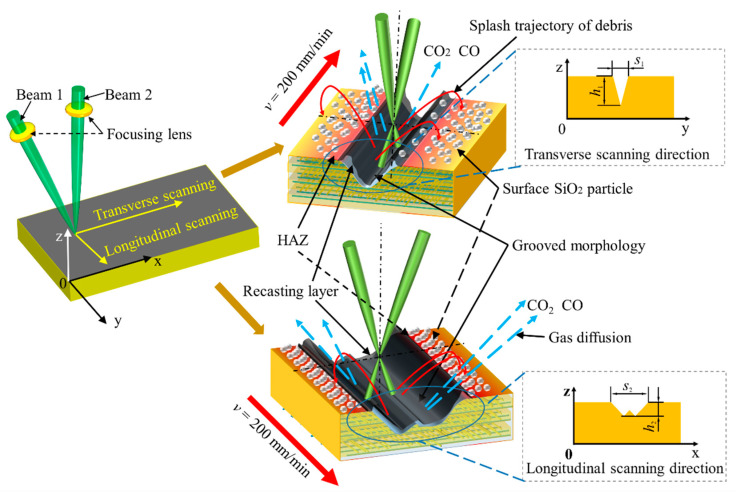
Schematic diagram of CMC-SiC_f_/SiC by using dual-beam coupling nanosecond laser.

When CMC-SiC_f_/SiC is melted at a high temperature, the material is oxidized to SiO_2_ and aggregates to form solids of granular. Under high temperature and high pressure, SiO_2_ particles are continuously sprayed outward from the laser action area and attached to the grooving area under the action of gravity to form the grooving affected area. CO_2_, CO, and other gases generated during the grooving process are diffused into the air. Under medium energy density, the grooved morphology is significantly affected by the thermal conductivity of fibers, and the grooved surface morphology is irregular. Under high energy density, there is a difference between transverse scanning and longitudinal scanning in the grooving section morphology. Compared with the longitudinal scanning, the depth *h* of the transverse scanning grooving surface is larger, and the width *s* is smaller. The grooved morphology of transverse scanning is V shape, and that of longitudinal scanning is W shape, as shown in Figure 19.

## 5. Conclusions

In this paper, two groups of grooves of CMC-SiC_f_/SiC are carried out by using a new dual-beam coupling nanosecond laser. The effects of processing parameters such as scanning direction, laser power, and laser repetition frequency on the grooved morphology were studied, and the material removal mechanism and grooved morphology in different regions of CMC-SiC_f_/SiC were analyzed. The following conclusions are drawn:

(1) The thermal conductivity of fiber has a significant effect on the local characteristics of the grooved morphology when using a medium energy density grooving, such as the laser energy density is 8.22 J/cm^2^. The obvious recasting layer is produced after the laser is applied to CMC-SiC_f_/SiC when using a high energy density laser grooving, which directly affects the grooved morphology. The thermal conductivity of fiber has a significant effect on the local characteristics of the grooved morphology when using a medium energy density grooving, such as the laser energy density being greater than or equal to 19.18 J/cm^2^. When the dual-beam coupling nanosecond laser irradiated to CMC-SiC_f_/SiC, a small portion of solid CMC-SiC_f_/SiC was directly converted to gas and sublimated to remove, and most of the solid CMC-SiC_f_/SiC was removed when heated;

(2) The depth of the transverse grooving is greater than that of the longitudinal grooving when the laser beam is transverse and longitudinal scanning, the maximum grooving depth is approximately 145.39 μm when the laser energy density is 76.73 J/cm^2^, and the minimum grooving depth is approximately 83.76 μm when the laser energy density is about 29.59 J/cm^2^. The plasma shielding effect affects the grooving depth of dual-beam coupling nanosecond lasers. The fiber can be removed when the energy density in the laser spot is small, and the laser beam is directly irradiated on SiC fiber, but the reflected laser cannot remove the fiber. Compared to SiC matrix, SiC fibers are more difficult to be removed by laser;

(3) The grooved morphology of transverse scanning is V shape, and that of longitudinal scanning is W shape. In the transverse scanning of the dual-beam coupling nanosecond laser, the rejections of the two beams incident from the cross-section of the groove and acting on the bottom of the grooves almost coincide, and one is in front of the other. This is because of the existence of a secondary grooving effect in time. During the longitudinal scanning, two laser beams of light incident from two directions of the cross-section of the slot act on both sides of the slot and are synchronized simultaneously. This is because there is no secondary grooving effect. Therefore, the grooving section morphology of transverse scanning has a larger depth and smaller width than that of longitudinal scanning when the laser parameters are the same.

## Figures and Tables

**Table 1:** Experiment parameters of nanosecond laser grooving (experiment 1, medium energy density grooving).

Parameter	Numerical Value	
Laser power, *P*/W	10	
Repetition frequency, *f*/kHz	20	
laser energy density, *φ*/(J/cm^2^)	8.22	
Scanning direction	Longitudinal	Transverse

**Table 2:** Experiment parameters of nanosecond laser grooving (experiment 2, high energy density grooving).

Parameter	Numerical Value					
Laser power, *P*/W	14	15	16		17	18
Repetition frequency, *f*/kHz	3	5	7		10	12
Scanning direction	Longitudinal			Transverse		

**Table 3:** The laser energy density corresponding to different laser powers when the repetition frequency is 10 kHz.

Laser Power, *P*/W	14	15	16	17	18
laser energy density, *φ*/(J/cm^2^)	23.01	24.66	26.31	27.95	29.59

**Table 4:** The laser energy density corresponding to different laser repetition frequencies when the laser power is 14 W.

Repetition Frequency, *f*/kHz	3	5	7	10	12
laser energy density, *φ*/(J/cm^2^)	76.73	46.04	32.88	23.01	19.18

## Data Availability

Not applicable.
